# Adherence to the ABC (atrial fibrillation better care) pathway and risk of adverse outcomes in patients with chronic kidney disease: a report from the prospective APHRS-AF registry

**DOI:** 10.1016/j.lanwpc.2025.101570

**Published:** 2025-05-12

**Authors:** Tommaso Bucci, Katarzyna Nabradalik, Krysztof Irlik, Alena Shantsila, Giulio Francesco Romiti, Marco Proietti, Wee-Siong Teo, Hyung-Wook Park, Wataru Shimizu, Hung-Fat Tse, Tze-Fan Chao, Gregory Y.H. Lip, APHRS-AF Registry Investigators, Hung-Fat Tse, Chun-Wah David Siu, Wataru Shimizu, Kenji Yodogawa, Hiroyuki Tsutsui, Yasushi Mukai, Hirofumi Tomita, Daisuke Horiuchi, Joji Hagii, Kazutaka Aonuma, Yasuo Okumura, Masahiko Goya, Kenzo Hirao, Ajioka Masayoshi, Nobuhisa Hagiwara, Atsushi Suzuki, Teiichi Yamane, Takanori Ikeda, Hitomi Yuzawa, Kazuhiro Satomi, Yoshinao Yazaki, Keiichi Fukuda, Yoshinori Kobayashi, Norishige Morita, Toyoaki Murohara, Eiichi Watanabe, Masahide Harada, Satoru Sakagami, Takahiro Saeki, Kengo Kusano, Koji Miyamoto, Shinsuke Miyazaki, Hiroshi Tada, Koichi Inoue, Nobuaki Tanaka, Yukihiro Koretsune, Haruhiko Abe, Yasuki Kihara, Yukiko Nakano, Akihiko Shimizu, Yasuhiro Yoshiga, Tomohiro Sakamoto, Ken Okumura, Naohiko Takahashi, Tetsuji Shinohara, Kyoko Soejima, Masahiko Takagi, Mitsuharu Kawamura, Yumi Munetsugu, Hyung-Wook Park, Jae-Min Shim, Jae Sun Uhm, Sung Il Im, Hyoung-Seob Par, Jun Hyung Kim, Young Keun On, Il-Young Oh, Seung Yong Shin, Jum Suk Ko, Jun Beom Park, Wee-Siong Teo, Kelvin Cheok-Keng Wong, Toon-Wei Lim, David Foo, Shih-Ann Chen, Tze-Fan Chao, YennJiang Lin, Fa-Po Chung, Yu-Feng Hu, Shil-Lin Chang, Ta-Chuan Tuan, Jo-Nan Liao, Cheng-Hung Li, Jin-Long Huang, Yu-Cheng Hsieh, Tsu-Juey Wu, Ying-Chieh Liao, Cheng-Hung Chiang, Hsiang-Chiang Hsiao, Tung-Chen Yeh, Wei-Siang Lin, Wen-Yu Lin, Jen-Yuan Kuo, Chong-Lie Hong, Yih-Je Wu, Ying-Siang Li, Jui-Peng Tsai, Kuo-Tzu Sung, Sheng-Hsiung Chang

**Affiliations:** a Liverpool Centre for Cardiovascular Science at University of Liverpool, Liverpool John Moores University and Liverpool and Heart and Chest Hospital, Liverpool, United Kingdom; b Department of Clinical Internal, Anesthesiologic and Cardiovascular Sciences, Sapienza University of Rome, Rome, Italy; c Department of Internal Medicine, Diabetology and Nephrology, Faculty of Medical Sciences in Zabrze, Medical University of Silesia, Katowice, Poland; d Department of Translational and Precision Medicine, Sapienza, University of Rome, Rome, Italy; e Department of Clinical Sciences and Community Health, University of Milan, Milan, Italy; f Division of Subacute Care, IRCCS Istituti Clinici Scientifici Maugeri, Milan, Italy; g Department of Cardiology, National Heart Centre, Singapore, Singapore; h Department of Cardiovascular Medicine, Chonnam National University Hospital, Gwangju, South Korea; i Department of Cardiovascular Medicine, Nippon Medical School, Tokyo, Japan; j Division of Cardiology, Department of Medicine, School of Clinical Medicine, Queen Mary Hospital, The University of Hong Kong, Hong Kong SAR, China; k Institute of Clinical Medicine and Cardiovascular Research Center, National Yang Ming Chiao Tung University, Taipei, Taiwan; l Division of Cardiology, Department of Medicine, Taipei Veterans General Hospital, Taipei, Taiwan; m Danish Center for Health Services Research, Department of Clinical Medicine, Aalborg University, Aalborg, Denmark; n Department of Cardiology, Lipidology and Internal Medicine, Medical University of Bialystok, Bialystok, Poland

**Keywords:** Atrial fibrillation, Cardiovascular events, Chronic kidney disease, ABC pathway

## Abstract

**Background:**

Limited data exist on the effectiveness of the ABC (Atrial Fibrillation Better Care) pathway in reducing adverse events in Asian patients with atrial fibrillation (AF) and chronic kidney disease (CKD).

**Methods:**

A post-hoc analysis of the prospective APHRS AF Registry. Patients were divided into CKD (eGFR < 60 ml/min) and non-CKD (eGFR ≥ 60 ml/min) groups. Logistic regression assessed factors associated with CKD, oral anticoagulant (OAC) use, and rhythm control strategies. Cox regression estimated hazard ratios (HRs) for a composite outcome of all-cause mortality and major adverse cardiovascular events. Subgroup analyses evaluated outcomes by CKD severity and ABC adherence.

**Findings:**

Of 3550 patients, 1029 had CKD (mean age 75.3 ± 10.3 years, 40.3% female), and 2521 did not (66.4 ± 11.3 years, 32.3% female). CKD patients were older, more often female, had lower ABC adherence (29.5% vs. 42.1%, p < 0.001) and anticoagulation use (Odds Ratio [OR] 0.77, 95% CI 0.61–0.96), but higher warfarin use, and were less likely to receive rhythm control (OR 0.79, 95% CI 0.66–0.94) comparing to those without CKD. CKD and adherence to the ABC pathway were independently associated with higher (HR 1.90, 95% CI 1.46–2.48) and lower (HR 0.64, 95% CI 0.48–0.87) risks of the composite outcome, respectively. Adverse event risks increased with CKD severity, and ABC pathway benefits were observed irrespective of CKD.

**Interpretation:**

AF patients with CKD show lower ABC pathway adherence and high risk of adverse events. Improving adherence to integrated care approaches may improve prognosis in this patient group.

**Funding:**

This study was an independent research grant by 10.13039/100004319Pfizer and Bristol Myers Squibb (BMS) to APHRS.

**Research in context**

**Evidence before this study**

The coexistence of atrial fibrillation (AF) and chronic kidney disease (CKD) presents a challenging clinical scenario, as these conditions share risk factors and mutually exacerbate adverse outcomes. Current international guidelines for AF management emphasize holistic and integrated approaches, such as the ABC (Atrial fibrillation Better Care) pathway, to tailor therapy to individual patient characteristics. However, data on the effectiveness of the ABC pathway in patients with AF and CKD, particularly within Asian populations, remain limited.

**Added value of this study**

This study demonstrates that the “clinical complexity” associated with CKD in AF patients can be effectively managed using the ABC pathway. By adopting this integrated and holistic approach, it is possible to carefully choose the most appropriate therapeutic treatment, considering how cardiovascular treatments may impact renal function.

**Implications of all the available evidence**

The concept underlying the ABC pathway can be further enhanced by systematizing other key aspects of AF management, such as optimal rate and rhythm control strategies based on the degree of renal impairment, alongside considering factors like ethnicity, diet, and environmental influences when assessing cardiovascular risk.

## Introduction

The management of patients with atrial fibrillation (AF) and chronic kidney disease (CKD) represents one of the most challenging clinical scenarios.^1^ These two conditions not only share several risk factors, including advanced age, hypertension, dyslipidaemia, obesity, and diabetes, but, when coexisting, exponentially increase the risk of adverse events.^2^ The mechanisms behind the increased risk of adverse events in patients with AF and CKD are complex, involving metabolic, pharmacokinetic, and vascular factors.^3^ This complexity underscores the need for tailored approaches that account for the bidirectional relationship between AF and CKD to assess the net clinical benefit of each decision.

A patient-centred, holistic or integrated care approach has been proposed to mitigate the risk of adverse events in patients with AF using the ABC (Atrial fibrillation Better Care) pathway.^4^ This approach includes three core principles crucial for addressing the clinical complexity of CKD in AF patients: appropriate oral anticoagulant (OAC) use, optimal rate or rhythm control, and comprehensive management of cardiovascular risk factors and comorbidities, as well as lifestyle modifications. Additionally, although adherence to the ABC pathway varies widely depending on the type of population selected and the geographical area considered,^5^ it has been associated with improved outcomes in AF patients, regardless of clinical complexity or educational level.^6^, ^7^, ^8^ This has led to its inclusion in guidelines globally, including those in Asia.^9^^,^^10^

Growing evidence indicates a substantial increase in the prevalence of CKD among Asians in recent years, with approximately 434.3 million individuals affected across Eastern, Southern, and Southeastern Asia, and up to 65.6 million experiencing advanced stages of the disease.^11^ Furthermore, research suggests that CKD may exert a more pronounced negative effect on the risk of adverse events in Asian patients compared to Western populations.^12^ However, data on the effectiveness of the ABC pathway in mitigating adverse events among patients with AF and CKD remain scarce,^13^^,^^14^ and specifically, its impact on Asian populations remains largely unexplored.

In 2015, the Asia–Pacific Heart Rhythm Society (APHRS) initiated a registry across five Asian geographical areas—Hong Kong, Singapore, South Korea, Japan, and Taiwan—to collect prospective data on the clinical progression of patients with AF.

The aims of this analysis were as follows: i) to identify clinical factors associated with CKD, ii) to examine differences in clinical management based on the presence of CKD, iii) to estimate the risk of adverse events in patients with AF and CKD, and iv) to evaluate adherence to the ABC pathway and its effectiveness in improving clinical outcomes for patients with CKD.

## Methods

The study protocol for patient enrolment and data collection followed the methodology of the ESC-European Heart Rhythm Association (EHRA) EURObservational Research Programme in AF General Long-Term (EORP-AF) Registry, as previously detailed.^7^ The study included consecutive inpatients and outpatients with AF who had undergone cardiology assessments in tertiary and general hospitals across five Asia–Pacific geographical areas (Hong Kong, South Korea, Japan, Singapore, and Taiwan). Enrolment began in 2015 and ended in 2017. All participants had an ECG confirming AF within one year prior to the initial visit and provided written informed consent, in compliance with the Declaration of Helsinki and local regulations. Following baseline assessments, local investigators performed a one-year follow-up.

### CKD definition

CKD was defined based on the estimated glomerular filtration rate (eGFR) calculated using the Modification of Diet in Renal Disease (MDRD) formula.^15^ Patients were classified as having CKD if their eGFR was <60 ml/min/1.73 m^2^. Furthermore, among individuals with CKD, an additional classification was made based on the degree of renal impairment: moderate CKD was defined as an estimated glomerular filtration rate (eGFR) between 30 and 59 ml/min/1.73 m^2^, while severe CKD was defined as an eGFR of less than 30 ml/min/1.73 m^2^.^16^

### Rhythm control definitions

After enrolment, patients who underwent rhythm control interventions, including electrical or pharmacological cardioversion, catheter ablation, or were prescribed antiarrhythmic drugs (Class Ia, Class Ic, Class III), were categorized into the “rhythm control” group. All other patients were classified as receiving “rate control” treatment strategies.

### Clinical scores

In the APHRS-AF Registry, OAC were prescribed according to the CHA_2_DS_2_-VASc score.^17^ Indication to OAC was made according to the AF guidelines that was utilized during the enrolment period (2015–2017).^18^^,^^19^ The HAS-BLED score was utilized to assess bleeding risk.^20^ Classification of AF-related symptoms was performed according to the EHRA score,^21^ as follows: EHRA I, no symptoms; EHRA II, mild symptoms (normal daily activity not affected); EHRA III, severe symptoms (normal daily activity affected); EHRA IV, disabling symptoms (normal daily activity discontinued). EHRA score considers symptoms attributable to AF and reverse or reduce upon restoration of sinus rhythm or with effective rate control and it was determined by recruiting sites.

Adherence to the ABC pathway was evaluated according to a previous analysis performed in the APHRS-AF Registry.^6^ In brief, each patient was considered compliant for:

• “A” Criterion: when properly prescribed with OAC according to the CHA_2_DS_2_-VASc score. OAC was considered as appropriate treatment in male patients with CHA_2_DS_2_-VASc ≥ 1 or female patients with CHA_2_DS_2_-VASc ≥ 2; patients not qualifying for OAC therapy (CHA_2_DS_2_-VASc = 0 in male patients or 1 in female patients) and not treated with OAC, also qualified for the “A” criterion.
• “B” Criterion: when reported an EHRA score of I or II.
• “C” Criterion: when all the following comorbidities associated with AF e.g., hypertension, coronary artery disease, peripheral arterial disease, heart failure, stroke, and diabetes mellitus were properly treated according to the current clinical guidelines. Optimal medical treatment for the C criterion was defined as follows: 1) hypertension: when blood pressure at baseline was less than 140/90 mmHg; 2) coronary artery disease: treatment included angiotensin-converting enzyme inhibitors, or angiotensin receptor blockers, beta blockers, and statins; 3) peripheral artery disease: treatment included statins; 4) previous stroke: treatment included statins; 5) heart failure: treatment comprised angiotensin-converting enzyme inhibitors, or angiotensin receptor blockers, and beta blockers; 6) diabetes mellitus: treatment included either insulin or oral glucose lowering agents.

Patients were considered adherent to the ABC pathway if they were adherent to all three criteria. All patients with at least 1 ABC criterion not attained were considered as ‘ABC non-adherent’. We also assessed adherence based on the number of ABC pathway criteria met.

### Outcomes

Adverse events were recorded throughout the one-year follow-up period. The primary outcome was a composite of all-cause death and major adverse cardiovascular events (MACE), which included cardiovascular death, thromboembolic events, acute coronary syndromes or significant coronary artery disease requiring percutaneous coronary interventional procedures, and new or worsening heart failure. Secondary outcomes included each component of the primary outcome. Finally, we conducted an exploratory analysis to investigate the risks associated with each MACE component individually.

### Statistical analysis

Categorical variables are presented as counts and percentages, while continuous variables are expressed as means ± standard deviation (SD) and compared using Student’s T-test. Proportions were compared with the χ^2^ test. Multivariable logistic regression was performed to evaluate: i) clinical factors associated with CKD, ii) the odds of receiving OAC, vitamin K antagonist anticoagulants (VKA) or non-vitamin K antagonist anticoagulants (NOAC), and iii) the likelihood of undergoing rhythm control strategies and ablation procedures, and iv) factors associated with adherence to the ABC pathway. The model utilised to identify factors associated with CKD was adjusted for age ≥ 75 years, female sex, paroxysmal AF, hypertension, vascular disease (defined as the presence of coronary artery disease or peripheral artery disease), heart failure, diabetes, previous stroke or transient ischaemic attack, cancer, chronic obstructive pulmonary disease (COPD), and history of bleeding. The models for rhythm control strategies and ABC pathway adherence were adjusted for the same variables, and CKD. All results from the logistic regression analyses were reported as odds ratio (OR), with 95% Confidence Interval (CI). Additionally, cubic spline curves were applied to the CHA_2_DS_2_-VASc score and eGFR to account for non-linear effects in relation to ABC pathway adherence. Predicted probabilities, along with 95% confidence intervals, were plotted to visualize these relationships. The incidence of adverse outcomes was calculated as the event count per total person-years and reported as the incidence per 100 person-years with relative 95% CI. Cox proportional hazards regression was employed for time-to-first-event analysis, estimating unadjusted and adjusted hazard ratios (HRs) and 95% CI for adverse events in patients with CKD compared to those without CKD. For the primary outcome, Kaplan–Meier curves were used to compare survival distributions, assessed using the log-rank test. The multivariable model for Cox regression analysis was adjusted for age ≥ 75 years, female sex, paroxysmal AF, CHA_2_DS_2_-VASc ≥ 2, COPD, cancer, and full ABC adherence. These variables were selected based on their potential association with the risk of the primary outcome, supporting our hypothesis that CKD is independently associated with a higher risk of adverse events and that adherence to the ABC pathway is an effective strategy to mitigate this risk.

Sensitivity analyses were performed to evaluate the risk of the primary outcome based on: i) CKD severity, and ii) the number of ABC criteria fulfilled. CKD severity was categorized as an ordinal variable with three levels: no or mild CKD, moderate CKD, and severe CKD. The number of ABC criteria fulfilled was categorized into three groups: 0 or 1 criterion, 2 criteria, and 3 criteria.

Moreover, we investigated the risk of the primary outcome based on the presence or absence of CKD and full adherence or non-adherence to the ABC pathway after propensity score matching (PSM). Propensity score matching was performed using logistic regression to balance the baseline characteristics of patients with and without CKD in a 1:1 ratio. The matching was conducted using the greedy nearest-neighbour method without a specific calliper. Absolute standardized mean differences (SMDs) were used to assess the distribution of demographic and clinical data among the groups and were calculated as the difference in the means or proportions of a given variable, divided by the pooled estimate of the standard deviation for that variable. Any baseline characteristic with an SMD < 0.1 was considered well-matched. For propensity score matching (PSM), we included the following variables: age class (<65, 65–74, ≥75 years), female sex, paroxysmal AF, hypertension, vascular disease, heart failure, diabetes, previous stroke or transient ischaemic attack, cancer, COPD, use of OAC, and history of bleeding. These variables were included due to their potential impact on the outcome of interest. A density plot was used to illustrate the propensity score before and after matching, while a Love plot was employed to display SMDs before and after matching. Univariable and multivariable Cox regression analyses were then performed. The multivariable Cox model was the same as that used for the main analyses.

Lastly, an interaction analysis was conducted to assess: i) the impact of CKD in clinically relevant subgroups (≥ or < 75 years, males or females, paroxysmal AF or other AF types, cancer or no cancer, COPD or no COPD, and CHA_2_DS_2_-VASc ≥ or < 2), and ii) the impact of the ABC adherence on reducing the risk of primary outcomes among patients with and without CKD.

Proportional hazard assumption for primary and secondary outcomes was tested using the Schoenfeld residuals test. The main analyses were performed with SPSS-29.0 software (SPSS Inc., Chicago, IL). Plots were generated with ggplot2 package, and models for spline curves were fitted using the glm() function with a binomial family. PSM was conducted using the MatchIt package in R version 4.3.1 (R Core Team, 2020, Vienna, Austria). Statistical significance was set at p-value < 0.05.

### Ethics approval

The study protocol was approved by the following coordinating centres: Taipei Veterans General Hospital (2016-10-005CC), University of Hong Kong (UW 16–196), Nippon Medical School Hospital (28-06-594), National Heart Centre Singapore (2016/2054), and Chonnam National University Hospital (CNUH-2016-331). The study was registered on ClinicalTrials.gov (NCT04807049).

### Role of the funding source

This study was supported by an independent research grant from Pfizer and Bristol Myers Squibb (BMS) to APHRS. The funders had no role in the study design, data collection, analysis, interpretation, or manuscript writing. No fees were received personally by the authors.

## Results

4666 patients with AF were enrolled on the APHRS-AF Registry, of whom 4121 (88.8%) were included in the prospective study. Of these, 3550 (86.1%) had complete data to assess renal function and adherence to the ABC pathway (Supplementary Fig. S1). Compared to patients included in this study, those excluded were younger, had a lower thrombotic and haemorrhagic risk, and had lower use of OAC, with no significant differences in sex prevalence (Supplementary Table S1).

### Clinical characteristics

The final cohort consisted of 2521 (71%) patients without CKD (mean age 66.4 ± 11.3 years, 32.3% female) and 1029 (29%) patients with CKD (mean age 75.3 ± 10.3 years, 40% female). Patients with CKD were older, more often females, and with a higher cardiovascular burden and haemorrhagic risk compared to those without CKD (Table 1). Compared to patients without CKD, those with CKD showed a similar use of OAC, but a higher use of VKA (Table 1). They were also less likely to receive rhythm control strategies, particularly ablation procedures. Patients with CKD had lower adherence to the “A” and “C” criteria and demonstrated significantly lower overall adherence to the ABC pathway than those without CKD (29.5% vs. 42.1%, p < 0.001; Table 1).

**Table 1:** Baseline characteristics of patients with and without chronic kidney disease.

	Patients without CKD n = 2521	Patients with CKD n = 1029	p-value
**Demographics**			
Age (years), mean ± SD	66.4 ± 11.3	75.3 ± 10.3	<0.001
Age < 65 years, n (%)	1019 (40.4)	152 (14.8)	
Age 65–74 years, n (%)	944 (37.4)	328 (31.9)	<0.001
Age ≥ 75 years, n (%)	558 (22.1)	549 (53.4)	
BMI k/m^2^	24.9 ± 4.2	25.3 ± 4.5	0.022
Female, n (%)	815 (32.3)	415 (40.3)	<0.001
**AF pattern, n (%)**			
First diagnosed	159 (6.3)	92 (8.9)	<0.001
Paroxysmal	1129 (44.9)	352 (34.2)	
Persistent	641 (25.5)	209 (20.3)	
Long standing persistent	209 (8.3)	114 (11.1)	
Permanent	374 (14.9)	262 (25.5)	
**Concomitant disease, n (%)**			
Hypertension	1418 (56.5)	801 (78.4)	<0.001
Vascular disease	438 (17.6)	311 (30.7)	<0.001
Heart failure	446 (17.8)	352 (34.7)	<0.001
Diabetes	539 (21.5)	369 (36.1)	<0.001
Dyslipidaemia	929 (37.2)	508 (50)	<0.001
Chronic obstructive pulmonary disease	62 (2.5)	44 (4.3)	0.004
Previous Stroke/TIA	216 (8.6)	142 (13.9)	<0.001
Previous bleedings	165 (6.6)	120 (11.7)	<0.001
Intracranial haemorrhage	37 (1.5)	31 (3.0)	0.004
Major extracranial bleeding	66 (2.6)	54 (5.3)	<0.001
Cancer	52 (2.1)	39 (3.8)	0.003
Dementia	30 (1.2)	38 (3.7)	<0.001
Anaemia	114 (4.5)	160 (15.6)	<0.001
**Medications, n (%)**			
ACE-I	303 (12.1)	194 (18.9)	<0.001
ARBs	636 (25.3)	321 (31.2)	<0.001
Beta blockers	1256 (50.0)	596 (58.0)	<0.001
Statins	910 (36.2)	513 (49.9)	<0.001
Digoxin	280 (11.1)	114 (11.1)	0.985
Diuretics	554 (22.0)	215 (21.0)	0.488
Aldosterone blockers	151 (6.0)	81 (7.9)	0.114
Calcium channel blockers	559 (22.3)	274 (26.7)	0.005
OAC	2146 (85.1)	860 (83.6)	0.245
VKA	447 (20.8)	282 (32.8)	<0.001
NOACs	1669 (79.2)	578 (67.2)	<0.001
Dabigatran	324 (12.9)	116 (11.3)	0.195
Rivaroxaban	602 (23.9)	183 (17.8)	<0.001
Apixaban	473 (18.8)	229 (22.3)	0.018
Edoxaban	300 (11.9)	50 (4.9)	<0.001
Antiplatelets	315 (12.5)	195 (19.0)	<0.001
**Thrombotic and haemorrhagic risk**			
CHA_2_DS_2_-VASc score, mean ± SD	2.4 ± 1.6	3.8 ± 1.6	<0.001
CHA_2_DS_2_-VASc ≥ 2	1708 (67.8)	957 (93.0)	<0.001
HAS-BLED, mean ± SD	1.2 ± 1.0	1.9 ± 1.1	<0.001
HAS-BLED ≥3	239 (9.5)	281 (27.3)	<0.001
**ABC pathway, n (%)**			
“A” adherence	2265 (89.8)	870 (84.5)	<0.001
“B” adherence	2341 (92.9)	974 (94.7)	0.051
“C” adherence	1253 (49.7)	367 (35.7)	<0.001
ABC pathway full adherence	1061 (42.1)	304 (29.5)	<0.001
**Rhythm control strategies, n (%)**			
Rhythm control approaches	1024 (40.8)	328 (32.0)	<0.001
Amiodarone	206 (8.2)	76 (7.4)	0.428
Dronedarone	64 (2.5)	27 (2.6)	0.886
Flecainide	118 (4.7)	40 (3.9)	0.297
Propafenone	199 (7.9)	62 (6.0)	0.052
Sotalol	42 (1.7)	19 (1.9)	0.709
Interventional procedures	599 (23.8)	148 (14.4)	<0.001
Electrical cardioversion	122 (4.8)	38 (3.7)	0.135
Pharmacological cardioversion	125 (5.0)	49 (4.8)	0.806
Ablation procedures	447 (17.7)	77 (7.5)	<0.001

ABC: Atrial fibrillation Better Care, ACE-I: Angiotensin-Converting Enzyme Inhibitors, ARBs: Angiotensin receptor blockers, BMI: Body Mass Index, CKD: Chronic Kidney Disease, OAC: Oral Anticoagulants, TIA: Transient Ischaemic attack, SD: Standard Deviation, VKA: Vitamin K antagonist anticoagulants, NOAC: Non vitamin K antagonist anticoagulants.

### Clinical factors associated with CKD

**Fig. 1:**
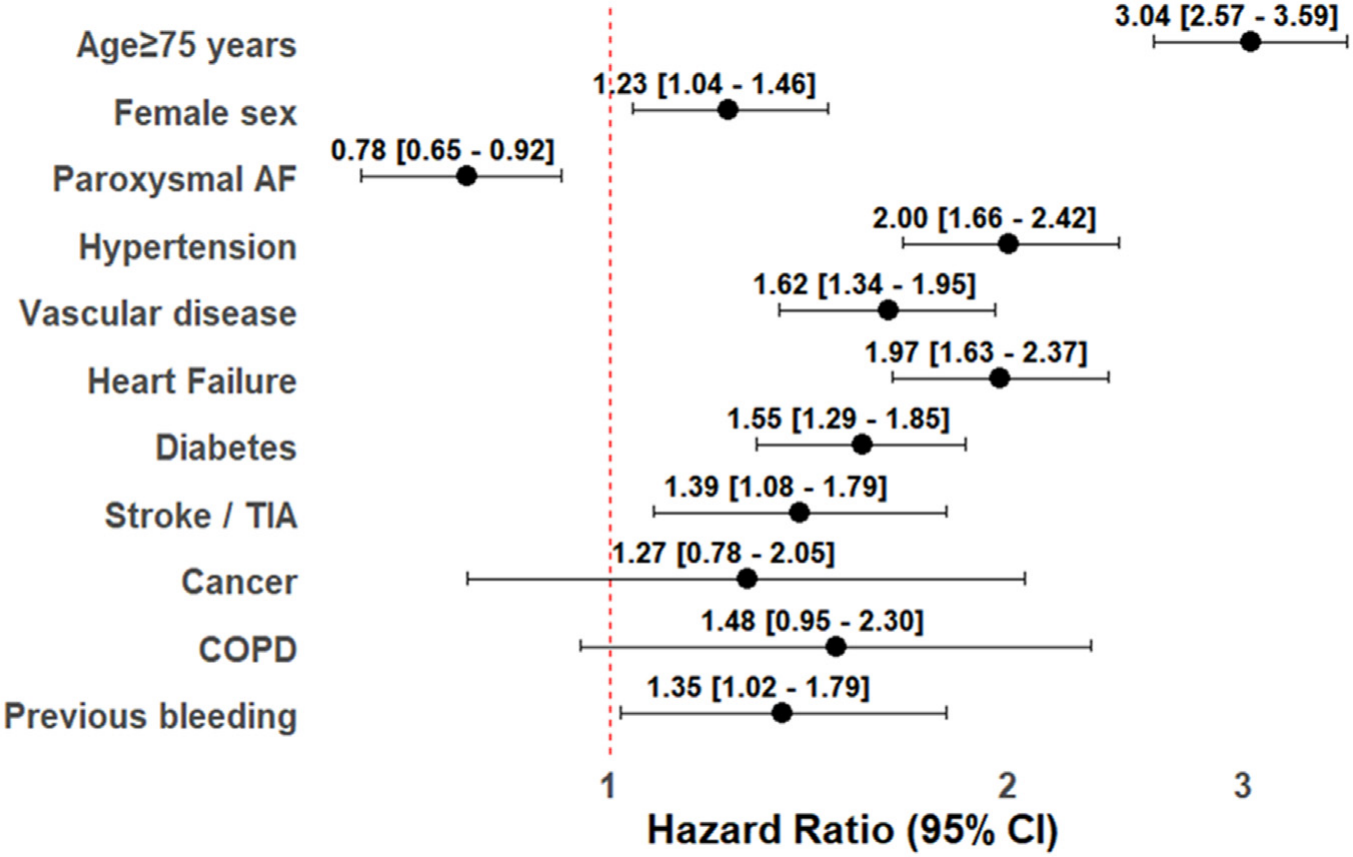
Clinical factors associated with chronic kidney disease on multivariable logistic regression analysis. Legend: AF: Atrial Fibrillation, CI: Confidence Intervals, COPD: Chronic Obstructive Pulmonary Disease, TIA: Transient ischaemic attack.

On multivariable logistic regression analysis, factors associated with CKD were advanced age, female sex, paroxysmal AF, hypertension, vascular disease, heart failure, diabetes, previous stroke, and history of bleeding. Non statistically significant associations were found for COPD and cancer (Fig. 1).

### Clinical management of patients with CKD

Multivariable logistic regression analyses showed that patients with CKD were less likely to receive OAC treatment (OR 0.77, 95% CI 0.61–0.96) and confirmed a lower use of rhythm control strategies (OR 0.79, 95% CI 0.66–0.94) (Table 2). When considering only patients on OAC or treated with rhythm control approaches, compared to those without CKD, patients with CKD had a higher use of VKA (OR 1.51, 95% CI 1.24–1.84) and a lower use of ablation procedures (OR 0.56, 95% CI 0.42–0.74), respectively (Table 2).

**Table 2:** Clinical factors associated with anticoagulant use and rhythm control strategies.

	Oral anticoagulant OR (95% CI)	VKA (vs. NOAC)^a^ OR (95% CI)
Age ≥ 75 years	1.04 (0.84–1.29)	0.88 (0.72–1.07)
Females	1.13 (0.92–1.39)	1.18 (0.98–1.42)
Paroxysmal AF	0.73 (0.60–0.86)	0.48 (0.39–0.58)
Hypertension	1.43 (1.17–1.75)	1.27 (1.04–1.55)
Vascular disease	0.86 (0.68–1.09)	1.33 (1.08–1.64)
Heart failure	1.16 (0.91–1.49)	1.49 (1.21–1.82)
Diabetes	1.11 (0.88–1.39)	1.18 (0.97–1.44)
Previous stroke/TIA	3.19 (2.02–5.05)	1.28 (0.98–1.67)
Cancer	0.65 (0.38–1.10)	0.41 (0.20–0.85)
COPD	0.84 (0.50–1.43)	1.29 (0.78–2.13)
Previous bleeding	0.48 (0.35–0.66)	0.96 (0.68–1.35)
Chronic kidney disease	0.77 (0.61–0.96)	1.51 (1.24–1.84)

	Rhythm control OR (95% CI)	Ablation procedures^b^ OR (95% CI)
Age ≥ 75 years	0.88 (0.75–1.04)	0.73 (0.57–0.95)
Females	0.41 (0.35–0.48)	0.06 (0.04–0.09)
Paroxysmal AF	0.83 (0.71–0.96)	1.01 (0.82–1.24)
Hypertension	0.98 (0.84–1.15)	0.70 (0.56–0.86)
Vascular disease	0.92 (0.77–1.11)	0.71 (0.54–0.94)
Heart failure	0.84 (0.70–1.01)	0.53 (0.39–0.72)
Diabetes	0.96 (0.81–1.14)	0.96 (0.75–1.24)
Previous stroke/TIA	1.05 (0.82–1.34)	0.68 (0.45–1.02)
Cancer	0.77 (0.47–1.24)	1.09 (0.55–2.15)
COPD	0.77 (0.50–1.19)	0.71 (0.38–1.33)
Previous bleeding	0.73 (0.55–0.97)	0.59 (0.36–0.96)
Chronic kidney disease	0.79 (0.66–0.94)	0.56 (0.42–0.74)

AF: Atrial Fibrillation, CI: Confidence Intervals, COPD: Chronic Obstructive Pulmonary Disease, NOAC: Non-vitamin k antagonist oral anticoagulants, OR Odds Ratio, TIA: Transient Ischaemic attack.

a Only in those on oral anticoagulants.

b Only in those on rhythm control.

### Clinical factors associated with ABC pathway adherence

**Fig. 2:**
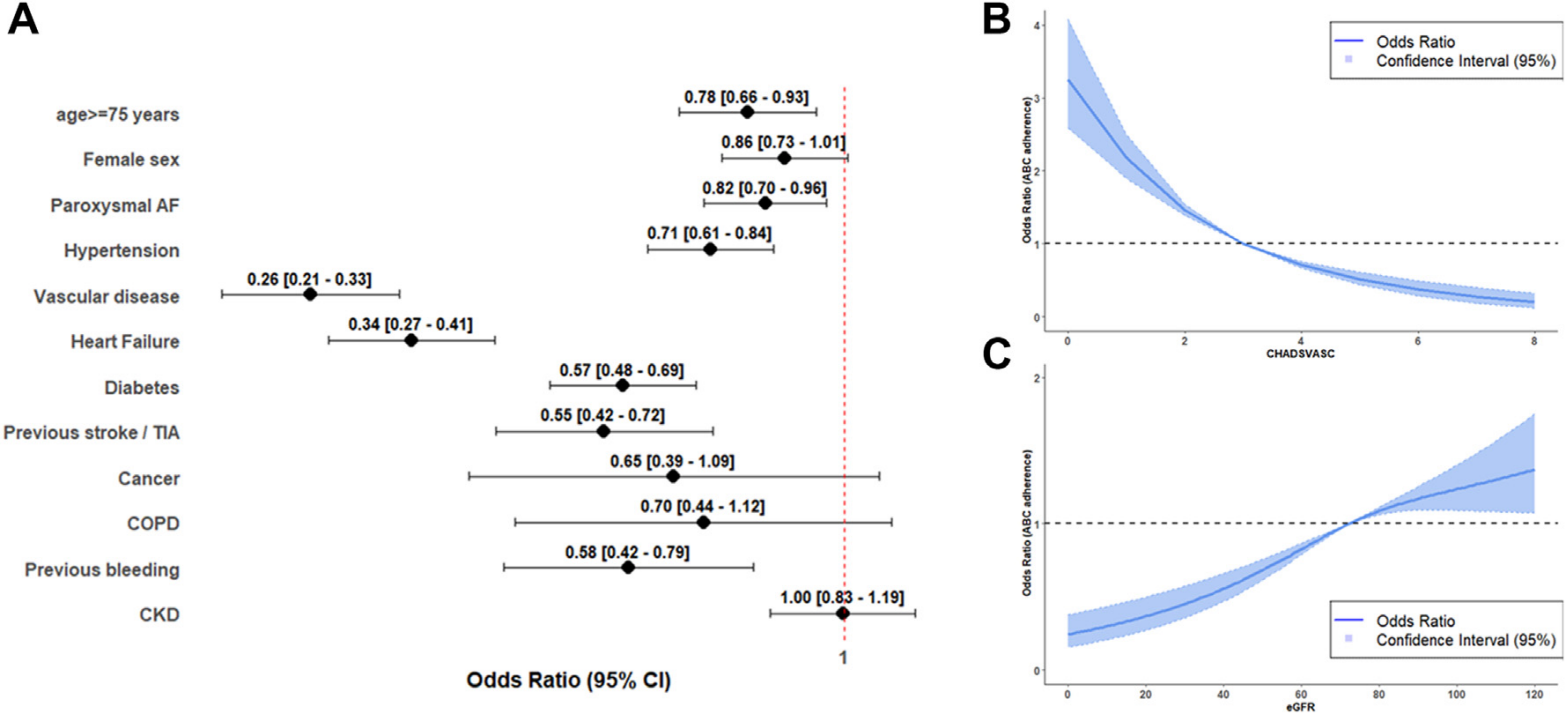
Multivariable logistic regression analysis for factors associated with ABC pathway adherence (Panel A), and spline curves showing changes in ABC adherence based on the CHA_2_DS_2_-VASc scores (Panel B) and estimated glomerular filtration rate (Panel C). Legend: AF: Atrial Fibrillation, CI: Confidence Intervals, CKD: Chronic Kidney Disease, COPD: Chronic Obstructive Pulmonary Disease, TIA: Transient ischaemic attack.

On multivariable regression analysis, factors associated with lower adherence to the ABC pathway included age ≥ 75 years, paroxysmal AF, hypertension, vascular disease, heart failure, diabetes, previous stroke, and previous bleeding (Fig. 2, Panel A). Non-statistically significant trends were observed for female sex, cancer, and COPD, while no association was found with CKD (Fig. 2, Panel A).

The strong relationship observed for clinical factors included in the CHA_2_DS_2_-VASc score was further supported by the dedicated spline curve, which demonstrated a linear inverse relationship between adherence to the ABC pathway and increasing CHA_2_DS_2_-VASc scores (Fig. 2, Panel B). Regarding the CKD, although no association was found on the multivariable model, a linear direct relationship was found between eGFR and the odds of being adherent to the ABC pathway (Fig. 2, Panel C).

### Survival analysis

**Fig. 3:**
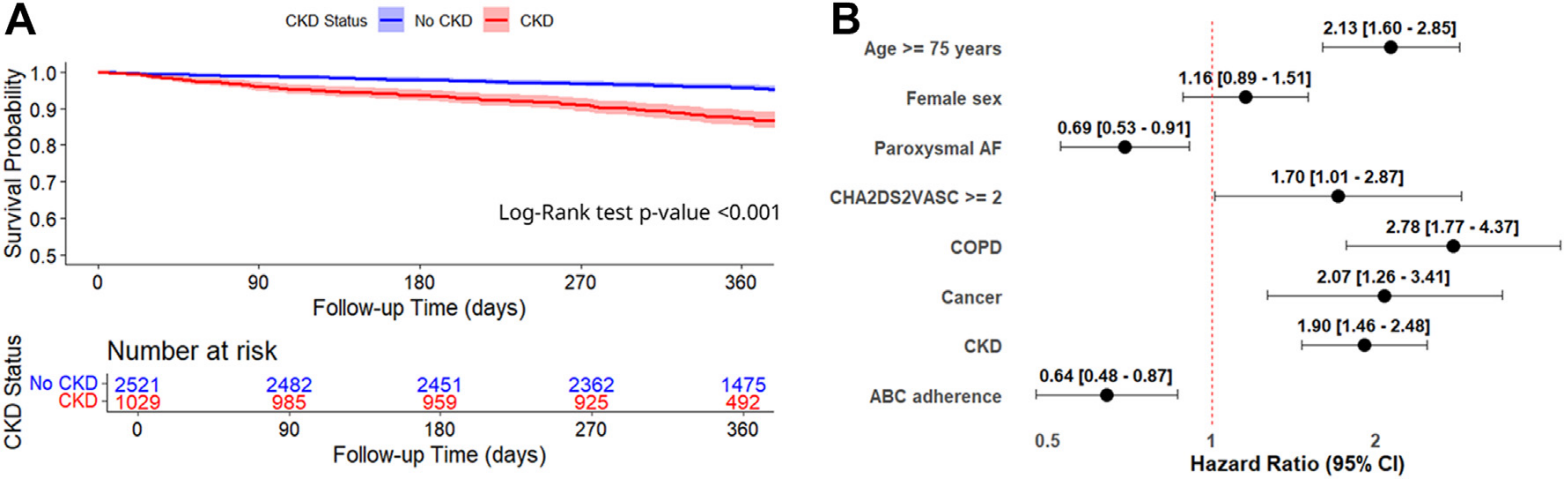
Kaplan–Meier curves showing the risk of the primary outcome in patients with and without chronic kidney disease (Panel A), and Cox multivariable analysis of the risk of the primary outcome (Panel B). Legend: ABC: Atrial fibrillation Better Care, AF: Atrial Fibrillation, CI: Confidence Interval, COPD: Chronic Obstructive Pulmonary Disease, CKD: Chronic Kidney Disease, HR: Hazard Ratio.

After one-year follow-up the following events were recorded: 245 (6.9%) composite outcome, 111 (3.1%) all-cause death, 159 (4.5%) MACE, 25 (0.7%) cardiovascular death, 38 (1.1%) acute coronary syndrome, 26 (0.7%) thromboembolic events, and 86 (2.4%) heart failure episodes. Compared to patients without CKD, those with CKD had a higher incidence of the composite outcome (Fig. 3, Panel A), all-cause death, MACE, cardiovascular death, and heart failure (Table 3). The higher risks for these outcomes were confirmed on univariable regression analyses (Table 3).

**Table 3:** Incidence rates and Cox regression analyses for risk of adverse events according to chronic kidney disease.

	Number of events	Incidence rate/100 persons/year (95% CI)	p-value	Univariable analysis HR (95% CI)	Multivariable analysis HR (95% CI)
**Composite outcome**					
No CKD	115	4.7 (3.8–5.6)	<0.001	Reference	Reference
CKD	130	13.7 (11.4–16.2)		3.00 (2.31–3.83)	1.90 (1.46–2.48)
**All-cause death**					
No CKD	39	1.6 (1.1–2.1)	<0.001	Reference	Reference
CKD	72	7.3 (5.7–9.2)		4.77 (3.23–7.05)	2.54 (1.69–3.80)
**MACE**					
No CKD	86	3.4 (2.8–4.3)	<0.001	Reference	Reference
CKD	73	7.5 (5.9–9.4)		2.24 (1.64–3.06)	1.58 (1.14–2.20)
**CV Death**					
No CKD	10	0.4 (0.2–0.7)	0.004	Reference	Reference
CKD	15	1.5 (0.9–2.5)		3.86 (1.73–8.60)	2.06 (0.91–4.68)
**ACS/PCI**					
No CKD	28	1.1 (0.7–1.6)	0.811	Reference	Reference
CKD	10	1.0 (0.5–1.9)		0.92 (0.45–1.89)	0.64 (0.30–1.36)
**Thromboembolic event**					
No CKD	15	0.6 (0.3–1.0)	0.097	Reference	Reference
CKD	11	1.1 (0.6–2.0)		2.04 (0.93–4.48)	1.42 (0.61–3.30)
**New or worsening HF**					
No CKD	41	1.6 (1.1–2.2)	<0.001	Reference	Reference
CKD	45	4.7 (3.4–6.3)		2.78 (1.82–4.24)	2.05 (1.30–3.21)

ACS: Acute Coronary Syndrome, CI: Confidence Intervals, CKD: Chronic Kidney Disease, CV: Cardiovascular, HF: Heart Failure, HR: Hazard Ratio, PCI: Percutaneous Coronary Interventional procedures, MACE: Major Adverse Cardiovascular Events.

On multivariable analysis, compared to patients without CKD, those with CKD had a higher risk of the composite outcome (HR 1.90, 95% CI 1.46–2.48, Fig. 3, Panel B), all-cause death (HR 2.54, 95% CI 1.69–3.80), and MACE (HR 1.58, 95% CI 1.14–2.20), with no violation of the proportional hazard assumption (Table 3, Supplementary Tables S2 and S3). Among the components of MACE, CKD was associated with a higher risk of heart failure, whereas only a trend was observed for cardiovascular death, and no statistically significant association was found with acute coronary syndrome or thromboembolic events compared to those without CKD (Table 3 and Supplementary Table S2).

In all analyses performed to assess the risk of primary and secondary outcomes, full ABC pathway adherence was associated with a significantly reduced incidence of both primary and secondary outcomes (Supplementary Table S4). When compared to patients who were non-adherent to the ABC pathway, those who were adherent had a lower risk of the composite outcome (HR 0.64, 95% CI 0.48–0.87) (Fig. 3), all-cause death (HR 0.57, 95% CI 0.35–0.93) (Supplementary Table S2), and MACE (HR 0.66, 95% CI 0.46–0.95) (Supplementary Table S2).

### Sensitivity analyses

When analysing the risk of the primary composite outcome based on CKD severity, a progressive increase in risk was observed from patients with moderate CKD (HR 1.52, 95% CI 1.14–2.03) to those with severe CKD (HR 4.66, 95% CI 3.20–6.78), compared to patients with no or mild CKD (Supplementary Table S5, Model A). However, even when considering different degrees of CKD severity, full adherence to the ABC pathway was still associated with a reduced risk of the composite outcome (HR 0.67, 95% CI 0.50–0.91) (Supplementary Table S5, Model A).

With respect to the number of ABC criteria fulfilled, a progressively reduced risk of the composite outcome was observed in patients meeting 2 criteria (HR 0.64, 95% CI 0.46–0.90) or 3 criteria (HR 0.45, 95% CI 0.31–0.67), compared to those meeting 0 or 1 criterion (Supplementary Tables S4 and S5, Model B). This demonstrates that an increasing number of ABC criteria fulfilled was associated with a progressively reduced risk of the composite outcome.

In the third sensitivity analysis, we included 2423 patients without CKD (21.8% aged ≥ 75 years, 32.2% females) and 983 patients with CKD (52.9% aged ≥ 75 years, 40% females) who had complete data for all variables included in the PSM (Supplementary Fig. S1 and Table S6).

**Fig. 4:**
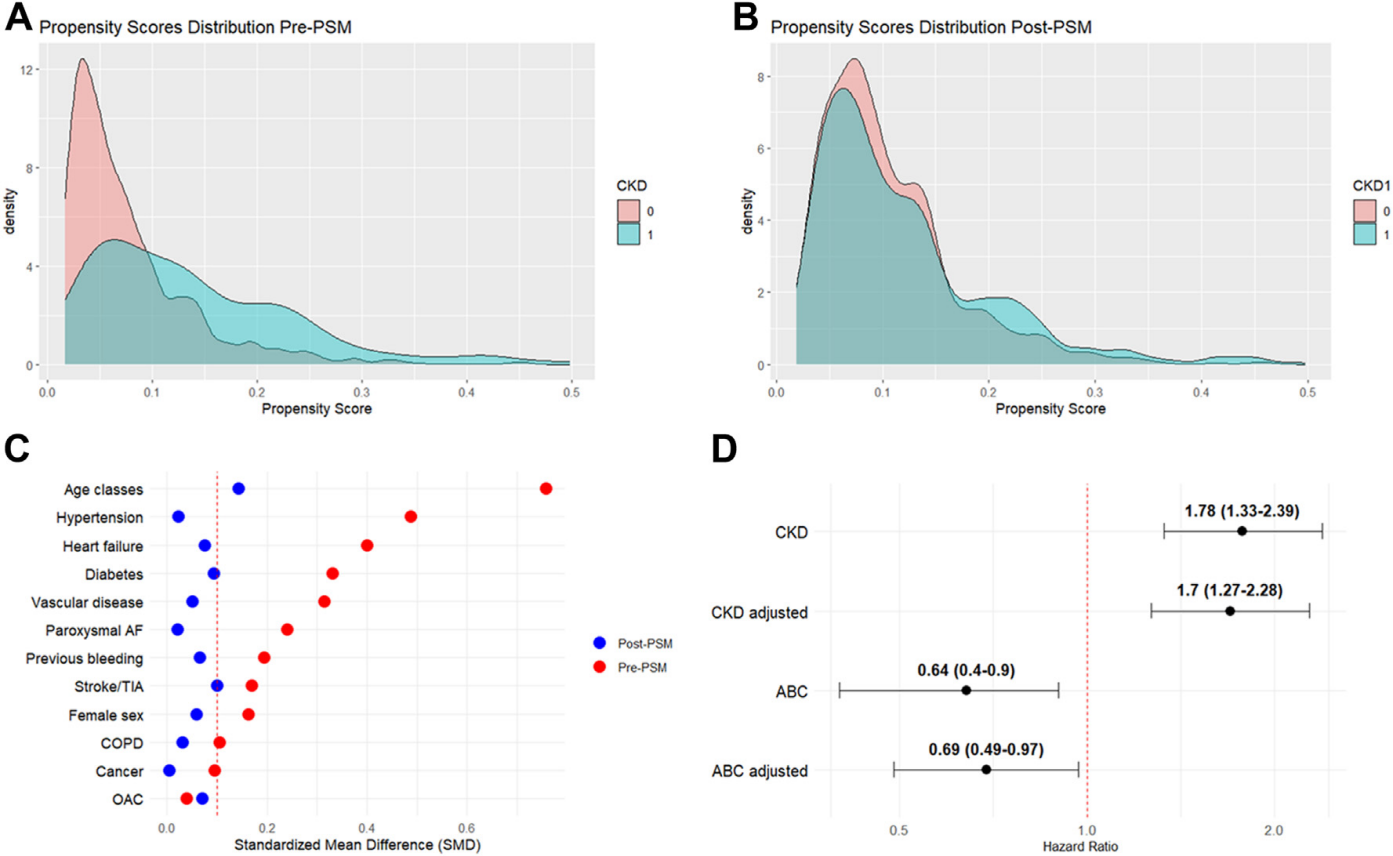
Distribution of propensity scores before (Panel A) and after (Panel B) matching, with corresponding changes in Absolute Mean Differences (Panel C) and results from univariable and multivariable Cox regression analyses after propensity score matching (Panel D).

Before PSM, AF patients with CKD were older and had a higher cardiovascular burden compared to those without CKD, but no difference was found in the use of OAC (Fig. 4 Panels A and C, and Supplementary Table S6). After PSM, 983 patients were included in each group, and no differences were found except for the age class ≥75 years, which was more prevalent in AF patients with CKD compared to those without (52.9% vs. 47.4%, respectively, SMD = 0.143), and prior stroke or TIA, which had an SMD at the threshold (13.7% vs. 10.5%, respectively, SMD = 0.100) (Fig. 4 Panels B and C, and Supplementary Table S6).

On multivariable Cox regression analysis after PSM, CKD was independently associated with an increased risk of the composite outcome (CKD: HR 1.70, 95% CI 1.27–2.28), whereas full ABC pathway adherence was associated with a significant protective effect (HR 0.69, 95% CI 0.49–0.97) (Fig. 4 Panel D, and Supplementary Table S7).

No violation of the proportional hazards assumption was found in either of the sensitivity analyses (Supplementary Table S3).

### Subgroup analyses

The detrimental effect of CKD on the risk of the composite outcome was observed irrespective of sex but appeared to be of greater magnitude in females compared to males (HR 2.29, 95% CI 1.50–3.48 vs. HR 1.62, 95% CI 1.14–2.29, respectively; p for interaction = 0.064) (Supplementary Table S8). The association between CKD and the composite outcome remained consistent across subgroups, including age, paroxysmal AF, COPD, cancer, and CHA_2_DS_2_-VASc ≥ 2, without significant differences (Supplementary Table S8).

The beneficial effect of full adherence to the ABC pathway was observed regardless of CKD presence or absence in both multivariable Cox models, performed before and after PSM (p-values for interaction = 0.762 and 0.482, respectively) (Supplementary Tables S9 and S10).

## Discussion

In this study from a prospective international registry of Asian patients, the main findings were as follows: i) AF patients with CKD exhibited a clinically complex phenotype, characterized by advanced age, female sex, high atherosclerotic burden, and a history of previous bleeding; ii) These patients were associated with a low use of OACs, relatively high use of VKAs, and a reduced likelihood of undergoing rhythm control strategies, particularly ablation procedures; iii) eGFR was directly associated with the likelihood of adherence to the ABC pathway; however, after adjusting for confounders, this relationship seemed to be mainly influenced by CKD-related factors incorporated in the CHA_2_DS_2_-VASc score rather than by CKD itself; iv) CKD was associated with a high risk of all-cause death and MACE during follow-up, with the risk increasing in patients with severe CKD; v) full adherence to the ABC pathway was associated with a reduced risk of adverse events, with a beneficial effects similarly observed in both patients with and without CKD.

The ‘clinically complex’ phenotype observed in patients with AF and CKD supports previous studies that show these two conditions often result from the presence of multiple cardiovascular risk factors and other comorbidities, which complicate the clinical course of preexisting conditions.^22^^,^^23^ Indeed, the onset of AF in patients with CKD has been associated with accelerated renal impairment, as well as an increased risk of hospitalization, thromboembolic events, and death.^24^, ^25^, ^26^ Similarly, the occurrence of CKD in patients with AF significantly reduces quality of life, limits options for OAC, and increases the risk of both thrombotic and haemorrhagic events.^26^, ^27^, ^28^

Moreover, our study confirms previous evidence indicating that female sex is associated with a higher risk of CKD compared to males. The 2017 Global Burden of Disease Study reported that the global age-adjusted prevalence of CKD in females was 9.5% (8.8%–10.2%), which was higher than the 7.3% (6.8%–7.9%) observed in males.^29^ Additionally, a large meta-analysis of 171 cohorts from 15 Asian countries, encompassing 2,550,169 females and 2,595,299 males, showed that the pooled prevalence of CKD was higher in females (13.0%, 95% CI 11.3–14.9) compared to males (12.1%, 95% CI 10.3–14.1).^30^ These sex related differences have been related with different factors. Females are more prone to developing autoimmune diseases with renal involvement,^31^ which consequently increases the risk of adverse events, particularly in those with AF.^32^ The decline in oestrogen during menopause may contribute to renal damage and seems to increase the susceptibility of women with diabetes and hypertension to renal involvement compared to men.^33^ Moreover, sex-related social disparities may further contribute to this risk,^34^^,^^35^ as well as AF-related complications such as stroke.^36^

In this study, after adjustment for confounders, we also corroborate the lower use of OAC among persons with CKD, described in previous reports.^37^ A substantial underuse of OAC has been noted in patients with AF and CKD, not only in those with end-stage renal disease but also in those with moderate renal impairment.^3^^,^^38^ Indeed, OAC is underutilized in these populations, primarily due to concerns about bleeding risks and challenges with renal dosing adjustments.^3^ This is further confirmed in our cohort, where the low use of OAC was associated with a relatively higher use of VKA in patients with CKD compared to those without, which may have amplified concerns regarding treatment safety and efficacy, despite evidence on the efficacy and safety of NOAC in patients with creatinine clearance down to 25 ml/min.^39^

This clinical scenario becomes even more complex when considering the impact of CKD on other key aspects of AF management. The presence of CKD was associated with a lower use of rhythm control strategies, particularly ablation procedures. Given recent studies indicating that rhythm control strategies are linked to better outcomes compared to those focused on controlling heart rate,^40^^,^^41^ this may partially explain the higher risk of adverse events observed in CKD patients in our cohort. Indeed, patients with AF and CKD face a greater risk of the composite outcome, as well as increased risk of all-cause death and MACE compared to those without CKD. This high risk of adverse events has been previously reported and linked to several mechanisms.^42^ CKD is associated with hyperkalaemia and metabolic acidosis, which promote muscle protein breakdown and impair myocardial cell function, leading to structural and electrical heart remodelling.^43^ Additionally, CKD accelerates atherosclerosis and increases vascular stiffness, predisposing patients to both thrombotic and haemorrhagic events.^44^ Reduced metabolism of renally cleared drugs may limit the safe use of OACs or antiarrhythmics, potentially exposing patients to unpredictable side effects.^45^^,^^46^ Furthermore, protein loss in skeletal muscle contributes to frailty, reducing performance status and increasing mortality risk beyond cardiovascular causes.^47^

Consistent with these pathophysiological changes, we found that the high risk of composite adverse events in AF patients with CKD was significantly associated with an increased risk of MACE, although being primarily driven by all-cause death. This highlights the need for holistic approaches to address the clinical complexities involved in managing AF in CKD patients. The choice of OAC in patients with CKD and AF should be based not only on eGFR but also on factors such as age, body mass index, concomitant treatments, exchange therapies, and the presence of specific immunosuppressive treatments following renal transplant, which may affect their plasma concentration and the duration spent within therapeutic ranges.^48^ Additionally, every decision should consider the effects of cardiovascular treatments on renal function.^49^ For example, aggressive blood pressure management with angiotensin-converting enzyme inhibitors, angiotensin receptor blockers, and diuretics may reduce renal blood flow and accelerate CKD progression.^50^ Therefore, since both organ systems are interrelated, therapeutic strategies must balance the benefits and potential side effects, carefully considering how cardiovascular treatments may impact renal function and vice versa.

In this study, adherence to the ABC pathway was associated with a 36% reduced risk of a composite outcome, including all-cause death and MACE. This finding aligns with the results of two post-hoc analyses—the ESC-EHRA EORP-AF long-term general registry^13^ and the SPORTIF III and V trials^14^—which reported a 49% and 55% reduced risk of a similar outcome in AF patients with CKD. Recently, recognizing the importance of the principles underlying the ABC pathway, both European and North American guidelines have promoted this approach, through the AF-CARE (Atrial Fibrillation: Comprehensive Assessment and Risk Evaluation) and S.O.S. (Stroke prevention, Optimization of all modifiable risk factors, and Symptom management) acronyms, respectively,^51^^,^^52^ although these new acronyms have not yet been investigated in clinical trials or real world cohorts.^53^ This shows that the holistic approach underlying the ABC pathway could be seen as an open-source model with limitless potential for further development. Indeed, there is room for improvement through the integration and systematization of additional aspects of clinical management for patients with AF and CKD. For instance, the optimal rhythm or rate control strategy should be tailored to the degree of renal impairment, and cardiovascular risk management could be enhanced by incorporating ethnic, dietary, social, and environmental factors.

Achieving this goal requires a comprehensive assessment of each patient’s comorbidities and an understanding of how each specific treatment interacts with others in order to determine the most effective approach with the best net clinical benefit. It is evident that as more comorbidities coexist, the integrated approach becomes increasingly complex. This complexity is underscored by our findings, which show that adherence to the ABC pathway decreases progressively as the number of comorbidities in the CHA_2_DS_2_-VASc score increases, making it more challenging to fulfil the C criteria and achieve full adherence to the ABC pathway.

Given the difficulties in developing a clinical algorithm that accounts for all possible combinations of demographic and clinical factors, along with the absence of evidence-based guidelines for certain conditions, the integration of artificial intelligence (AI) and digital twins may provide valuable support.^54^, ^55^, ^56^ For example, the ARISTOTELES (Applying ARtificial Intelligence to Define clinical trajectorieS for personalized predicTiOn and early deTEctiOn of comorbidiTy and muLti-morbidiTy pattErnS) project, a multicentre study involving 18 European health institutions, aims to provide novel insights.^55^ ARISTOTELES will create a global platform that integrates clinical records, biomarkers, imaging, and genetic data from diverse real-world sources. This harmonized data will train AI models to learn from varied patient populations and disease conditions. These AI models will be tested in clinical trials to generate actionable insights for improving ABC pathway adherence and evaluating its effectiveness in complex real-world scenarios. The project aims to develop personalized algorithms that support individualized treatment strategies within the ABC pathway framework.

### Limitations

This study has several limitations. As an observational non-randomised study, causality cannot be inferred due to the potential for unmeasured biases. The post-hoc analysis design may have introduced additional confounding factors that were not accounted for in the original study design. The study population represents only a subset of the broader Asian population, and racial differences in AF-related outcomes, such as stroke and bleeding, are evident.^57^, ^58^, ^59^ Caution is needed when generalizing the results of this study to the entire Asian population. The rhythm control approaches were defined based on the type of therapeutic treatment prescribed, with no data provided regarding their efficacy. Most importantly, adherence to the A criterion was not tailored to the dosage for those on NOACs or to the time spent within the therapeutic range for those on VKAs. This may have led to the misclassification of some patients as appropriately treated with OACs. Some residual bias may have occurred due to the exclusion of patients without follow-up or those with missing data. Additionally, no data on major bleeding risks related to CKD were provided, offering only a partial view of the adverse event risks in these patients. The study period predates recent evidence supporting the safety and efficacy of NOACs in patients with end-stage renal disease,^60^ which may have resulted in a higher likelihood of VKAs being prescribed to more severely ill patients, potentially influencing the risk of adverse events in CKD patients. Moreover, CKD phenotype related to albuminuria was not examined due to lack of data and no analysis was conducted on the association between CKD-related adverse events and social determinants of health.

### Conclusions

Patients with AF and CKD exhibit a distinctive clinical phenotype and had an increased risk of adverse events. Integrated approaches that account for the broad clinical heterogeneity of CKD are essential to minimizing the risk of adverse events in these patients.

## Contributors

TB: conceptualisation, formal analysis, writing original draft; KN, KI, AS, GFR, MP: review & editing; WST, HWP, WS, HFT investigation, review & editing; TFC and GHYL: conceptualisation, supervision, validation, and writing original draft. All authors approved the final version of the manuscript. TB, TFC, and GHYL have directly accessed and verified the underlying data. TB, TFC, and GHYL have directly accessed and verified the underlying data.

## Data sharing statement

Data underlying this study will be made available upon reasonable request to the corresponding authors.

## Declaration of interests

GFR reports consultancy for Boehringer Ingelheim and an educational grant from Anthos, outside the submitted work. No fees are directly received personally. MP is national leader of the AFFIRMO project on multimorbidity in AF, which has received funding from the European Union’s Horizon 2020 research and innovation program under grant agreement No 899871. WS has received grants from Daiichi Sankyo Co., Ltd. and Nippon Boehringer Ingelheim Co., Ltd.; and remuneration for lectures, presentations, speakers’ bureaus, manuscript writing, or educational events from Daiichi Sankyo Co., Ltd., Nippon Boehringer Ingelheim Co., Ltd., Bristol-Meyers Squibb, Bayer Yakuhin, Ltd., Pfizer Japan, Inc., Ono Pharmaceutical Co., Ltd., and Medtronic Japan Co., Ltd. HFT: is a consultant/speaker fee and research grants from for Abbott; Amgen; AstraZeneca; Bayer; BMS, Boehringer Ingelheim; Boston Scientific; Daiichi Sankyo; Medtronic; Novartis; Pfizer and Sanofi. GYHL has been a consultant and speaker for BMS/Pfizer, Boehringer Ingelheim, Anthos, and Daiichi-Sankyo. No fees are directly received personally. All the disclosures happened outside the submitted work. GYHL is a National Institute for Health and Care Research (NIHR) Senior Investigator and co-PI of the AFFIRMO project on multimorbidity in AF (grant agreement No 899871), TARGET project on digital twins for personalized management of atrial fibrillation and stroke (grant agreement No 101136244), and ARISTOTELES project on artificial intelligence for management of chronic long-term conditions (grant agreement No 101080189), which are all funded by the EU’s Horizon Europe Research and Innovation program.
