# Supplementary material for: Adherence to the ABC (atrial fibrillation better care) pathway and risk of adverse outcomes in patients with chronic kidney disease: a report from the prospective APHRS-AF registry
Source: Lancet Reg Health West Pac. 2025 May 12;58:101570. doi: 10.1016/j.lanwpc.2025.101570 (PMC12136897; doi:10.1016/j.lanwpc.2025.101570)
Supplement: Supplementary Fig. S1 and Tables S1–S10 [file mmc1.docx]

**Adherence to the ABC (Atrial fibrillation Better Care) pathway and risk of adverse outcomes in patients with chronic kidney disease: A report from the prospective APHRS-AF Registry.**

Tommaso Bucci, Katarzyna Nabradalik, Krysztof Irlik, Alena Shantsila, Giulio Francesco Romiti, Marco Proietti, Wee-Siong Teo, Hyung-Wook Park, Wataru Shimizu, Hung-Fat Tse, Tze-Fan Chao, Gregory Y. H. Lip.

Supplementary materials

Supplementary Figure 1. Flow diagram of the study.


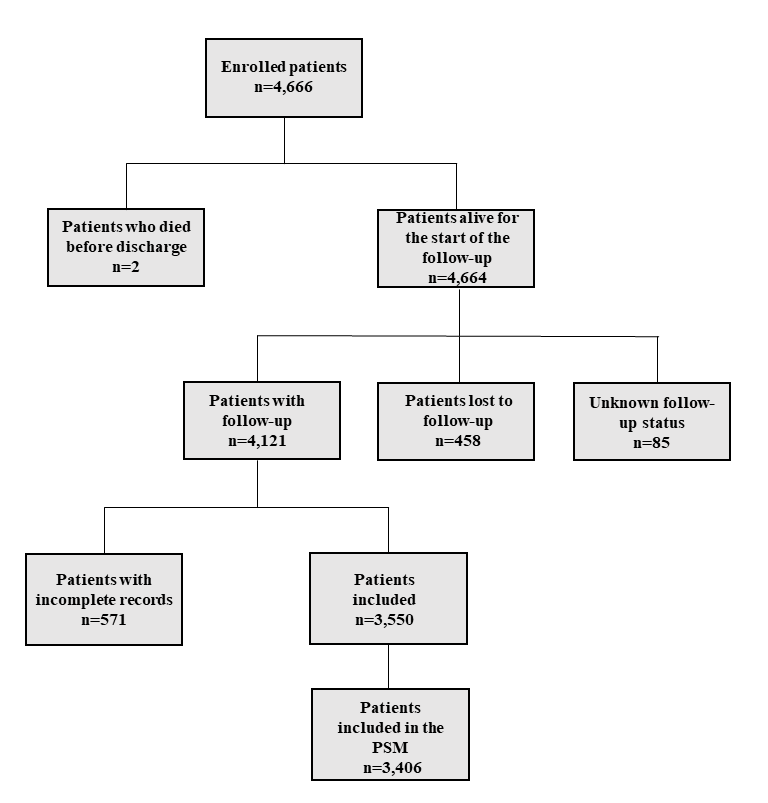


PSM: Propensity Score Matching.

Supplementary Table 1. Main characteristics of patients included in the study compared to those excluded

|  | Patients  excluded  (n=571) | Patients  included  (n=3550) | p-value |
| --- | --- | --- | --- |
| Age, years (mean ± SD) | 65.5±12.3 | 69.0±11.7 | <0.001 |
| Female sex, n (%) | 193 (33.8) | 1230 (34.6) | 0.717 |
| CHA_2_DS_2_-VASc ≥2, n (%) | 348 (60.9) | 2665 (75.1) | <0.001 |
| HAS-BLED ≥3, n (%) | 47 (8.2) | 520 (14.6) | <0.001 |
| OAC, n (%) | 396 (69.4) | 3006 (84.7) | <0.001 |

CKD: Chronic Kidney Disease, OAC: Oral Anticoagulants, SD: Standard Deviation

Supplementary Table 2. Multivariable models for the risk of secondary and exploratory outcomes.

|  | All-cause death  HR (95%CI) | MACE  HR (95%CI) | CV death  HR (95%CI) | ACS/PCI  HR (95%CI) | Thromboembolism  HR (95%CI) | Heart failure  HR (95%CI) |
| --- | --- | --- | --- | --- | --- | --- |
| Age≥75 years | 3.44 (2.13-5.55) | 1.65 (1.17-2.34) | 2.47 (1.01-6.07) | 1.87 (0.94-3.70) | 2.25 (0.89-5.68) | 1.50 (0.93-2.41) |
| Female sex | 1.17 (0.80-1.73) | 1.14 (0.82-1.58) | 1.27 (0.57-2.84) | 0.65 (0.32-1.30) | 1.94 (0.85-4.42) | 1.22 (0.78-1.91) |
| Paroxysmal AF | 0.55 (0.35-0.84) | 0.76 (0.54-1.05) | 0.41 (0.15-1.09) | 1.35 (0.71-2.56) | 0.87 (0.39-1.93) | 0.52 (0.32-0.84) |
| CHA_2_DS_2_-VASc ≥2 | 2.79 (0.82-9.47) | 1.73 (0.98-3.08) | * | 3.75 (1.06-13.31) | 0.83 (0.23-3.01) | 1.26 (0.60-2.67) |
| COPD | 3.60 (2.02-6.44) | 1.97 (1.02-3.79) | 2.19 (0.50-9.63) | 0.66 (0.09-4.89) | 1.47 (0.19-11.27) | 2.64 (1.19-5.85) |
| Cancer | 3.30 (1.84-5.90) | 0.97 (0.40-2.37) | 1.01 (0.14-7.52) | ° | 1.34 (0.18-9.98) | 1.09 (0.34-3.48) |
| CKD | 2.54 (1.69-3.80) | 1.58 (1.14-2.20) | 2.06 (0.91-4.68) | 0.64 (0.30-1.36) | 1.42 (0.61-3.30) | 2.05 (1.30-3.21) |
| ABC adherence | 0.57 (0.35-0.93) | 0.66 (0.46-0.95) | 0.44 (0.15-1.29) | 0.76 (0.37-1.53) | 0.65 (0.27-1.58) | 0.77 (0.48-1.24) |

ABC: Atrial fibrillation Better Care, ACS: Acute Coronary Syndrome, AF: Atrial Fibrillation, CI: Confidence Intervals, COPD: Chronic Obstructive Pulmonary Disease, CKD: Chronic Kidney Disease, CV: Cardiovascular, HR: Hazard Ratio, PCI: Percutaneous Coronary Interventional Procedures.

*No CV deaths were recorded in patients with CHA_2_DS_2_-VASc <2 without CKD.

°No ACS/PCI were recorded in patients without cancer and CKD.

Supplementary Table 3. Test for proportional hazard assumptions.

|  | **χ2** | **p-value** |
| --- | --- | --- |
| **Main analysis** |  |  |
| Composite outcome | 6.7 | 0.570 |
| MACE | 7.7 | 0.477 |
| All-cause death | 7.6 | 0.570 |
| **Sensitivity for CKD severity** |  |  |
| Composite outcome | 6.9 | 0.645 |
| **Sensitivity for number of ABC criteria** |  |  |
| Composite outcome | 7.5 | 0.580 |
| **Multivariable Cox regression after PSM** |  |  |
| Composite outcome | 3.9 | 0.862 |

ABC: Atrial fibrillation Better Care, CKD: Chronic Kidney Disease, MACE: Major Adverse Cardiovascular Events, PSM: Propensity Score Matching.

Supplementary Table 4. Incidence rates and Cox regression analyses for risk of adverse events according to ABC pathway adherence.

|  | **Number of events** | **Incidence rate / 100 persons / year (95%CI)** | **p-value** |
| --- | --- | --- | --- |
| **Composite outcome** | | | |
| Non-ABC adherent (n=2185) | 187 (8.6) | 9 (7.7-10.4) | <0.001 |
| ABC adherent (n=1365) | 58 (4.2) | 4.3 (3.3-5.6) |  |
| **Composite outcome** | | | |
| 0-1 ABC criterion fulfilled (n=376) | 48 (12.8) | 13.8 (10.2-18.3) | 0.001*  <0.001** |
| 2 ABC criteria fulfilled (n=1809) | 139 (7.7) | 8.0 (6.7-9.5) |  |
| 3 ABC criteria fulfilled (n=1365) | 58 (4.2) | 4.3 (3.3-5.6) | <0.001*** |
| **All-cause death** |  |  |  |
| Non-ABC adherent (n=2185) | 90 | 4.2 (3.4-5.2) | <0.001 |
| ABC adherent (n=1365) | 21 | 1.5 (0.9-2.3) |  |
| **MACE** |  |  |  |
| Non-ABC adherent (n=2185) | 118 | 5.7 (4.7-6.8) | <0.001 |
| ABC adherent (n=1365) | 41 | 2.6 (1.1-4.1) |  |

*0-1 ABC criterion vs 2 ABC criteria, **0-1 ABC criterion vs 3 ABC criteria, ***2 ABC criteria vs 3 criteria

ABC: Atrial fibrillation Better Care, ACS: Acute Coronary Syndrome, CI: Confidence Intervals, CKD: Chronic Kidney Disease, CV: Cardiovascular, HF: Heart Failure, HR: Hazard Ratio, PCI: Percutaneous Coronary Interventional procedures, MACE: Major Adverse Cardiovascular Events.

Supplementary Table 5. Sensitivity analyses for the risk of the composite primary outcome.

|  | Model A  HR (95%CI) | Model B  HR (95%CI) |
| --- | --- | --- |
| Age≥75 years | 2.12 (1.58-2.83) | 2.12 (1.58-2.83) |
| Female sex | 1.09 (0.83-1.42) | 1.15 (0.88-1.50) |
| Paroxysmal AF | 0.66 (0.50-0.87) | 0.68 (0.52-0.89) |
| CHA_2_DS_2_-VASc ≥2 | 1.75 (1.04-2.94) | 1.70 (1.01-2.86) |
| COPD | 2.86 (1.82-4.49) | 2.71 (1.73-4.27) |
| Cancer | 1.91 (1.16-3.15) | 1.93 (1.17-3.18) |
| No or mild CKD | Reference | - |
| Moderate CKD | 1.52 (1.14-2.03) | - |
| Severe CKD | 4.66 (3.20-6.78) | - |
| ABC pathway adherence | 0.67 (0.50-0.91) | - |
| CKD | - | 1.87 (1.45-2.46) |
| 0 or 1 ABC criterion | - | Reference |
| 2 ABC criteria | - | 0.64 (0.46-0.90) |
| 3 ABC criteria | - | 0.45 (0.31-0.67) |

ABC: Atrial fibrillation Better Care, AF: Atrial Fibrillation, CI: Confidence Intervals, COPD: Chronic Obstructive Pulmonary Disease, CKD: Chronic Kidney Disease, HR: Hazard Ratio,

Supplementary Table 6. Clinical characteristics of patients without and with CKD before and after propensity score matching.

|  | Before PSM | | | After PSM | | |
| --- | --- | --- | --- | --- | --- | --- |
|  | Patients without CKD  (n=2423) | Patients with  CKD  (n=983) | SMD | Patients without CKD  (n=983) | Patients with  CKD  (n=983) | SMD |
| <65 years, n (%) | 994 (41.0) | 150 (15.3) | 0.758 | 994 (41.0) | 150 (15.3) | 0.143 |
| 65-74 years, n (%) | 900 (37.1) | 313 (31.8) |  | 380 (38.7) | 313 (31.8) |  |
| ≥75 years, n (%) | 529 (21.8) | 520 (52.9) |  | 466 (47.4) | 520 (52.9) |  |
| Female sex, n (%) | 781 (32.2) | 393 (40.0) | 0.162 | 365 (37.1) | 393 (40.0) | 0.059 |
| Paroxysmal AF, n (%) | 1092 (45.1) | 328 (33.4) | 0.241 | 338 (34.4) | 328 (33.4) | 0.021 |
| Hypertension, n (%) | 1365 (56.3) | 772 (78.5) | 0.488 | 763 (77.6) | 772 (78.5) | 0.022 |
| Vascular disease, n (%) | 424 (17.5) | 303 (30.8) | 0.315 | 280 (28.5) | 303 (30.8) | 0.051 |
| Heart failure, n (%) | 412 (17.0) | 336 (34.2) | 0.401 | 301 (30.6) | 336 (34.2) | 0.076 |
| Diabetes, n (%) | 515 (21.3) | 354 (36.0) | 0.331 | 311 (31.6) | 354 (36.0) | 0.093 |
| Stroke/TIA, n (%) | 204 (8.4) | 135 (13.7) | 0.170 | 103 (10.5) | 135 (13.7) | 0.100 |
| Cancer, n (%) | 50 (2.1) | 36 (3.7) | 0.096 | 35 (3.6) | 36 (3.7) | 0.005 |
| COPD, n (%) | 58 (2.4) | 42 (4.3) | 0.105 | 36 (3.7) | 42 (4.3) | 0.031 |
| Previous bleeding, n (%) | 150 (6.2) | 115 (11.7) | 0.194 | 95 (9.7) | 115 (11.7) | 0.066 |
| OAC, n (%) | 2057 (84.9) | 820 (83.4) | 0.040 | 845 (86.0) | 820 (83.4) | 0.071 |

AF: Atrial Fibrillation, COPD: Chronic Obstructive Pulmonary Disease, CKD: Chronic Kidney Disease, OAC: Oral Anticoagulant, SMD: absolute Standardized Mean Difference, TIA: Transient Ischaemic Attack.

Supplementary Table 7. Multivariable Cox regression analysis after propensity score matching.

|  | HR (95%CI) |
| --- | --- |
| Age≥75 years | 1.82 (1.32-2.51) |
| Female sex | 1.15 (0.85-1.54) |
| Paroxysmal Atrial Fibrillation | 0.66 (0.48-0.91) |
| CHA_2_DS_2_VASc≥2 | 1.32 (0.59-2.92) |
| COPD | 3.00 (1.86-4.84) |
| Cancer | 1.99 (1.15-3.43) |
| CKD | 1.70 (1.27-2.28) |
| ABC pathway adherence | 0.69 (0.49-0.97) |

ABC: Atrial fibrillation Better Care, CKD: Chronic Kidney Disease, CI: Confidence Interval, COPD: Chronic Obstructive Pulmonary Disease. HR: Hazard Ratio.

Supplementary Table 8. Subgroups analyses for the risk of primary outcome in patients with chronic kidney disease.

|  | HR (95%CI) | p-value for interaction |
| --- | --- | --- |
| Age ≥75 years | 1.81 (1.30-2.51) | 0.644 |
| Age <75 years | 2.00 (1.29-3.12) |  |
| Males | 1.62 (1.14-2.29) | 0.064 |
| Females | 2.29 (1.50-3.48) |  |
| Paroxysmal AF | 1.63 (1.00-2.65) | 0.790 |
| No Paroxysmal AF | 2.01 (1.46-2.76) |  |
| COPD | 1.42 (0.59-3.39) | 0.455 |
| No COPD | 1.95 (1.48-2.58) |  |
| Cancer | 2.03 (0.73-5.71) | 0.881 |
| No Cancer | 1.90 (1.44-2.49) |  |
| CHA_2_DS_2_-VASc ≥2 | 1.86 (1.42-2.44) | 0.686 |
| CHA_2_DS_2_-VASc <2 | 2.28 (0.73-7.13) |  |

AF: Atrial Fibrillation, CI: Confidence Interval, COPD: Chronic Obstructive Pulmonary Disease, HR: Hazard Ratio.

Supplementary Table 9. Subgroup analyses of the risk of the primary outcome in patients adherent to the ABC pathway, with and without CKD, before PSM.

|  | HR (95%CI) | p-value for interaction |
| --- | --- | --- |
| No CKD | 0.61 (0.39-0.95) | 0.762 |
| CKD | 0.69 (0.46-1.04) |  |

ABC: Atrial fibrillation Better Care, AF: Atrial Fibrillation, CI: Confidence Interval, CKD: Chronic Kidney Disease, HR: Hazard Ratio, PSM: Propensity Score Matching.

Supplementary Table 10. Subgroup analyses of the risk of the primary outcome in patients adherent to the ABC pathway, with and without CKD, after PSM.

|  | HR (95%CI) | p-value for interaction |
| --- | --- | --- |
| No CKD | 0.80 (0.47-1.37) | 0.482 |
| CKD | 0.63 (0.40-0.97) |  |

ABC: Atrial fibrillation Better Care, AF: Atrial Fibrillation, CI: Confidence Interval, CKD: Chronic Kidney Disease, HR: Hazard Ratio. PSM: Propensity Score Matching.
